# Extrahepatic 25-Hydroxylation of Vitamin D_3_ in an Engineered Osteoblast Precursor Cell Line Exploring the Influence on Cellular Proliferation and Matrix Maturation during Bone Development

**DOI:** 10.1155/2013/956362

**Published:** 2013-06-04

**Authors:** Shelley S. Mason, Sean S. Kohles, Shelley R. Winn, Randy D. Zelick

**Affiliations:** 1 Department of Biology, Portland State University, P.O. Box 751, Portland, OR 97207-0751, USA; 2 Department of Molecular and Medical Genetics, Oregon Health & Science University, Portland, OR 97239, USA

## Abstract

Osteoblastic precursors experience distinct stages during differentiation and bone development, which include proliferation, extracellular matrix (ECM) maturation, and ECM mineralization. It is well known that vitamin D plays a large role in the regulation of bone mineralization and homeostasis via the endocrine system. The activation of vitamin D requires two sequential hydroxylation steps, first in the kidney and then in the liver, in order to carry out its role in calcium homeostasis. Recent research has demonstrated that human-derived mesenchymal stem cells (MSCs) and osteoblasts can metabolize the immediate vitamin D precursor 25-dihydroxyvitamin D_3_ (25OHD_3_) to the active steroid l*α*,25-dihydroxyvitamin D_3_ (1,25OH_2_D_3_) and elicit an osteogenic response. However, reports of extrahepatic metabolism of vitamin D_3_, the parental vitamin D precursor, have been limited. In this study, we investigated whether osteoblast precursors have the capacity to convert vitamin D_3_ to 1,25OH_2_D_3_ and examined the potential of vitamin D_3_ to induce 1,25OH_2_D_3_ associated biological activities in osteoblast precursors. It was demonstrated that the engineered osteoblast precursor derived from human marrow (OPC1) is capable of metabolizing vitamin D_3_ to 1,25OH_2_D_3_ in a dose-dependent manner. It was also demonstrated that administration of vitamin D_3_ leads to the increase in alkaline phosphatase (ALP) activity associated with osteoblast ECM maturation and calcium deposits and a decrease in cellular proliferation in both osteoblast precursor cell lines 0PC1 andOMC3T3-E1. These findings provide a two-dimensional culture foundation for future three-dimensional engineered tissue studies using the OPC1 cell line.

## 1. Introduction

An osteoblastic precursor cell line (OPC1), derived from human fetal bone tissue, was originally established to provide a consistent and reproducible culture system for evaluating bone development, cell/biomaterial interactions, and screening putative bone differentiating factors [1]. In addition, OPC1 can be used to study the growth and differentiation of osteoprogenitors and offers the possibility of examining events associated with stem cell differentiation to osteoblasts. Proliferation and differentiation of osteoblasts are regulated by their respective microenvironment, which encompasses cells, growth factors and cytokines, and their extracellular matrix (ECM) [2]. Osteoblasts are a main target for calcitropic hormones including vitamin D [3, 4]. Several studies have demonstrated changes in bone development in response to the steroid hormone vitamin D, especially with regard to the inhibition of growth during the proliferation stage and the stimulation of osteogenic differentiation during the maturation and mineralization stages, albeit the outcomes appear to be variable and dependent upon the stage of cellular maturation or differentiation at the time of hormone responsiveness [5, 6]. Because OPC1 is a stable osteoblast precursor that has displayed consistent reproducible cultures, it is an ideal culture system to study the effects of vitamin D on bone development and elucidate the extraendocrine metabolism of vitamin D in osteoblasts.

Vitamin D, also known as calciferol, refers to a collective group of fat-soluble pluripotent, secosteroid metabolites that together carry out the known vitamin-D-related functions. The classic understanding of vitamin D is the essential role it plays in the regulation and maintenance of calcium and phosphorous homeostasis and bone metabolism. Elucidating the steps required for the metabolism of vitamin D precursors, vitamin D_3_ (cholecalciferol), and 25-hydroxyvitamin D_3_ (25OHD_3_; calcidiol) to the active hormone 1*α*,25-dihydroxyvitamin D_3_ (1,25OH_2_D_3_; calcitriol) led to the discovery of regulatory cytochrome P-450 (CYP-related hydroxylases) involved in the activation and inactivation of vitamin D and the nuclear vitamin D receptor (VDR) and its interactions with transcriptional machinery inside vitamin D target cells [7]. 1,25OH_2_D_3_ directly influences osteoblast binding to the VDR, which consequently elicits nuclear and extranuclear cellular responses related to the production and maintenance of the skeleton [3, 4]. Once it was demonstrated that VDR and vitamin D regulatory CYPs were present in multiple tissues and cells not involved in the classic endocrine actions of vitamin D, it was acknowledged that its functions extended far beyond mineral homeostasis and bone metabolism. It is now recognized that vitamin D is associated with the regulation of many cellular processes including proliferation, differentiation, and apoptosis in multiple tissues in a paracrine/autocrine manner [7–9].

Vitamin D, in the form of vitamin D_3_, is endogenously made from cholesterol in the skin when it is exposed to ultraviolet light (UVB). Alternatively, vitamin D can be acquired by diet in either the vitamin D_3_ (derived from animals) or vitamin D_2_ (derived from plants) form. There are many metabolites or isomers of vitamin D. The active metabolite, 1,25OH_2_D_3_, and the immediate metabolic precursor to the active metabolite, 25OHD_3_, are among the most studied. Vitamin D_3_, the metabolic precursor to 25OHD_3_, itself is biologically inert, or inactive, in that it does not directly increase the active transport of calcium or mediate any of the other vitamin-D-related activities in physiological concentrations unless it is metabolized to a more polar compound [10]. In the classic endocrine metabolic pathway of vitamin D, both endogenous vitamin D_3_ and dietary vitamin D_3_ undergo sequential steps of activation by being hydroxylated in the liver to 25OHD_3_ by CYP-related 25-hydroxylase (CYP27A1) and then to 1,25OH_2_D_3_ by 25-hydroxyvitamin D_3_-1*α* -hydroxylase (CYP27B1) in the kidneys [7, 11, 12]. While early data suggested that the liver is the only significant site of 25-hydroxylation *in vivo*, there have been occasional reports of extrahepatic 25-hydroxylation of vitamin D_3_ [7, 13]. Recently, extrarenal metabolism of 25OHD_3_ to 1,25OH_2_D_3_ has been demonstrated definitively [9, 14]. *In vitro*, many nonrenal cells, including mesenchymal stem cells (MSCs), bone, cartilage, keratinocytes, placenta, prostate, macrophages, lymphocytes, dendritic cells, and several cancer cell lines, can convert 25OHD_3_ to 1,25OH_2_D_3_ [14]. Several of these cell types have been shown to express both CYP27 hydroxylase enzymes required for the activation of vitamin D_3_, which may explain the occasional reports of extrahepatic 25-hydroxylation of vitamin D_3_ [7, 13, 15].

The molecular effects of vitamin D are mediated through the intranuclear receptor VDR, which acts as a ligand-activated transcription factor for target genes when bound to 1,25OH_2_D_3_ [16]. 1,25OH_2_D_3_ has a high affinity for VDR and upon binding forms a heterodimer with the retinoid X receptor, which allows it to bind to the response/receptor element (VDRE) in the promoter regions of target genes [8, 17, 18]. The genes include those associated with calcium homeostasis, immune response and maintenance of immune cells, cellular growth, differentiation, cell cycle arrest, apoptosis, and the enzymes required for their own metabolism [17–20]. 1,25OH_2_D_3_ influences many aspects of bone cell biology and has been implicated in the regulation of both osteoblastic and osteoclastic activity affecting both resorptive and synthetic phases of bone remodeling [21]. In addition, 1,25OH_2_D_3_ has been demonstrated to regulate osteoblast and chondrocyte gene transcription, proliferation, differentiation, and mineralization of ECM [20, 21]. More than 30 tissues are known to have nuclear receptors for 1,25OH_2_D_3_, and it is known to regulate the function of more than 60 genes [11, 13, 17, 22]. Because of its role in proliferation and differentiation, it has been hypothesized that the local production of 1,25OH_2_D_3_ in extrarenal tissues serves to regulate growth and differentiation in an autocrine/paracrine fashion [21, 23].

Despite the increase in research over the last few decades, further investigation on vitamin D metabolism and regulation needs to be carried out in order to elucidate extraendocrine metabolism and local production of 1,25OH_2_D_3_. While there has been an increase in the reports of extrarenal 1,25OH_2_D_3_ synthesis and occasional reports of extrahepatic 25-hydroxylation of vitamin D_3_, to our knowledge, there is no detailed analysis of extrahepatic vitamin D_3_ metabolism in osteoblast precursors. Furthermore, paracrine/autocrine regulation and gene expression that result in local changes in proliferation and differentiation need to be further investigated to understand the potential application of vitamin D for regenerative medicine. In this study, we investigated whether osteoblast precursors have the capacity to convert the parental vitamin D precursor, vitamin D_3_, to 1,25OH_2_D_3_ and examined the potential of vitamin D_3_ to induce 1,25OH_2_D_3_ associated biological activities in osteoblast precursors.

## 2. Materials and Methods

### 2.1. OPC1 and MC3T3-E1 Cell Cultures.

Vitamin D_3_ (chole-calciferol) and 1,25OH_2_D_3_ (calcitriol) were purchased and maintained in 10 mM and 10 *μ*M stock solutions in ethanol, respectively (Sigma-Aldrich, St. Louis, MO). All other reagents and chemicals were purchased from the same vendor unless otherwise indicated. Original OPC1 and MC3T3-E1 cell lines were cultured on site (Department of Molecular & Medical Genetics, Oregon Health & Science University, Portland, OR).

Cells were cultured at populations of 2.5 × 10^5^ in 75 cm^2^ tissue culture flasks in alpha modified essential medium (*α*-MEM) with 5% fetal bovine serum (FBS). Once confluent, OPCs were plated in 12-well plates after trypsin-ethylene-diaminetetraacetic acid (EDTA) enzymatic removal and counted with 0.4% trypan blue (1: 1) on a hemacytometer. Cultures were then prepared in duplicate at a seeding density of 2.5 × 10^4^ cells/well in the initial bone medium (designated as BM−) with 200 mM L-Glutamine and antibiotics for at least 4 hours before creating experimental groups based on additive culture medium. Six types of medium-based groups were prepared. BM− provided a negative control (Group 1) and the additional groups included BM− supplemented with 1 *μ*M vitamin D_3_ (Group 2); BM− supplemented with 10 nM 1,25OH_2_D_3_ (Group 3); BM− supplemented with osteogenic factors (BM+) comprised of 10 mM *β*-glycerophosphate, 10 nM dexamethasone, and 50 *μ*g/mL of ascorbic acid phosphate (Wako Chemical, Osaka, Japan) represented the positive control (Group 4); BM+ supplemented with 1 μM vitamin D_3_ (Group 5); and BM+ supplemented with 10 nM 1,25OH_2_D_3_ (Group 6). Fresh medium was added every 1 to 3 days.

### 2.2. Cell Proliferation.

OPC1 and MC3T3-E1 cell lines were evaluated for growth kinetics while maintained in the presence of vitamin D metabolites, vitamin D_3_ and 1,25OH_2_D_3_, with (BM+) or without (BM−) osteogenic factors, at the indicated experimental group concentrations. Each sample set was cultured for at least two weeks in duplicate or quadruplicate. Every 2 to 5 days, following the addition of experimental media and at the end of each incubation period, cell proliferation was measured using crystal violet solution (CVS, Sigma-Aldrich). Cultures were rinsed with Dulbecco’s phosphate buffered saline (DPBS) and fixed for 15 minutes in 95% ethanol. The fixed cells were rinsed several times with distilled water and stained for 10 minutes with CVS. The CVS was then extracted from the cells after a 4 h incubation in 1% Triton X-100 at room temperature. The samples were then stored at -80°C until the end of each experimental period. Triton extracts were measured at 570 nm on a microplate reader (Cary 50, Varian Australia Pty. Ltd.). Absorbance values were converted into cell numbers extrapolated from standard growth curves.

### 2.3. Alkaline Phosphatase.

As described above, OPC1 and MC3T3-E1 were treated with both vitamin D_3_ and 1,25OH_2_D_3_ in the BM− and BM+ experimental groups. Alkaline phosphatase (ALP) activity was measured every 3 to 4 days. At the end of the incubation, the cell layers were washed three times with DPBS, scraped off of the plates with 500 *μ*L DPBS, and stored at −80°C until the end of each experimental period (2 to 3 weeks). Each sample was subjected to three freeze-thaw cycles in order to lyse the cells. ALP activity in the cell lysates was measured using *ρ*-nitrophenyl phosphate liquid substrate system at 37°C for 30 minutes. Protein content was measured using a microvolume spectrophotometer system (Epoch, Biotek, Winooski, VT).

### 2.4. Matrix Mineralization.

A histochemical analysis of mineralization was evaluated utilizing an Alizarin Red S (ARS) assay. ARS staining is used to evaluate calcium-rich deposits by cells in culture and is considered a functional *in vitro* endpoint reflecting advanced cell differentiation [24]. In brief, after 10 to 15 days in culture, cells were fixed in 95% ethanol for 15 minutes then rinsed thoroughly with distilled water and stained with 40 mM of ARS (pH = 4.1) at room temperature for 20 min with gentle shaking. The cell layers were efficiently rinsed with distilled water and observed both grossly and microscopically. For semi-quantification, ARS was extracted from the cells after incubating for one hour at room temperature in 10% volume to volume (v/v) acetic acid with gentle shaking, and then the residual dye was further extracted by scraping the cells and heating at 85°C on a heating block with a layer of mineral oil to prevent evaporation. The samples were neutralized with 10% (v/v) ammonium hydroxide, and the extracts were read at a resolution of 405nm on the microplate reader. The samples were compared to a serially diluted ARS standard. The resulting data were normalized using cell numbers extrapolated from the CVS proliferation data.

### 2.5. Vitamin D_3_ Metabolism.

Vitamin D metabolism by OPC1 was performed in a dose-dependent manner over a 24-hour period. The conversion of 1,25OH_2_D_3_ from vitamin D was quantitatively measured using an enzyme-linked immunosorbent assay (ELISA) kit (Human 1,25-dihydroxyvitamin D_3_ DVD/DHVD3, NB-E10878, Novatein Biosciences, Cambridge, MA). OPC1 was plated in 6-well plates at a seeding density of 10^5^ cells/well. Upon confluency, experimental media were added in duplicates when creating experimental groups slightly modified from those described in Section 2.1: base medium (BM−) provided a negative control (Group 1); BM− supplemented with 10 nM 1m25OH_2_D_3_ as a positive control (Group 2); BM− supplemented with 10 nM vitamin D_3_ (Group 3); BM− supplemented with 1 *μ*M vitamin D_3_(Group 4); and BM− supplemented with 10 *μ*M vitamin D_3_ (Group 5). The assay was carried out via manufacturer’s protocol.

### 2.6. Statistical Analysis and Mathematical Modeling

Statistical analyses were carried out using commercial software (Prism, Irvine, CA; JMP Pro v10, SAS Concepts, Inc., Cary, NC; Excel, Microsoft, Redmond, WA). Data were expressed as means ± standard error of the mean (SEM) of samples characterized in three two-week sample sets plated in duplicate or quadruplicate and assayed in duplicate (*n* = 12 or *n* = 24, resp.). One-way and two-way analysis of variance (ANOVA) and linear regression statistically assessed the culture treatments through time and between experimental groups, with asterisks of *P < 0.05, **P < 0.01, and ***P < 0.001 indicating varying levels of statistical significance. Bonferonni posttest (direct comparisons with controls) and Dunnett’s test (pairwise comparisons) were applied where appropriate.

Through the ELISA assay protocol for examining the dose-response of the metabolized form of vitamin D, the input dosage levels (*x* -values) were controlled and distributed at logarithmic concentration levels (in units of ng/mL). A log-base-10 model was applied to the resulting response data (*y*-values in pmol/mg protein) in the form:

(1) y=mLog10x.

Here, the coefficient (*m*) was identified as a fitting parameter optimizing the model’s representation of the collected data. The model was applied to the metabolized vitamin D concentration data. Least squares estimates were constructed for the log-coefficient via a quasi-Newton convergence method with commercially available software (Solver, Frontline Systems, Inc., Incline Village, NV; Excel, Microsoft, Redmond, WA). The model fit was again determined by minimizing the root mean square error (RMSE) between the actual and response concentrations. The correlation coefficient (*R*^2^) further assayed the strength of the modeled relationship.

### 2.7. Microscopic Imaging.

Microscopic images were taken using an advanced transmitted light inverted microscope EVOS-XL) and accompanying software (EVOS3), both commercially available (Advanced Microscopy Group, Bothell, WA). Images were processed using an open source, image-processing package (Fiji, ImageJ v1.47d, National Institutes of Health, Bethesda, MD). To maintain consistency, each image was processed in the same manner including maintaining consistent light intensities during optical density measurements. The operations included the background subtraction command, mean filter, and local contrast (CLAHE commands: block size 100, bin 256, and maximum slope 2.50, resp.).

## 3. Results

### 3.1. ALP Activity and Early Matrix Mineralization.

**Figure 1:**
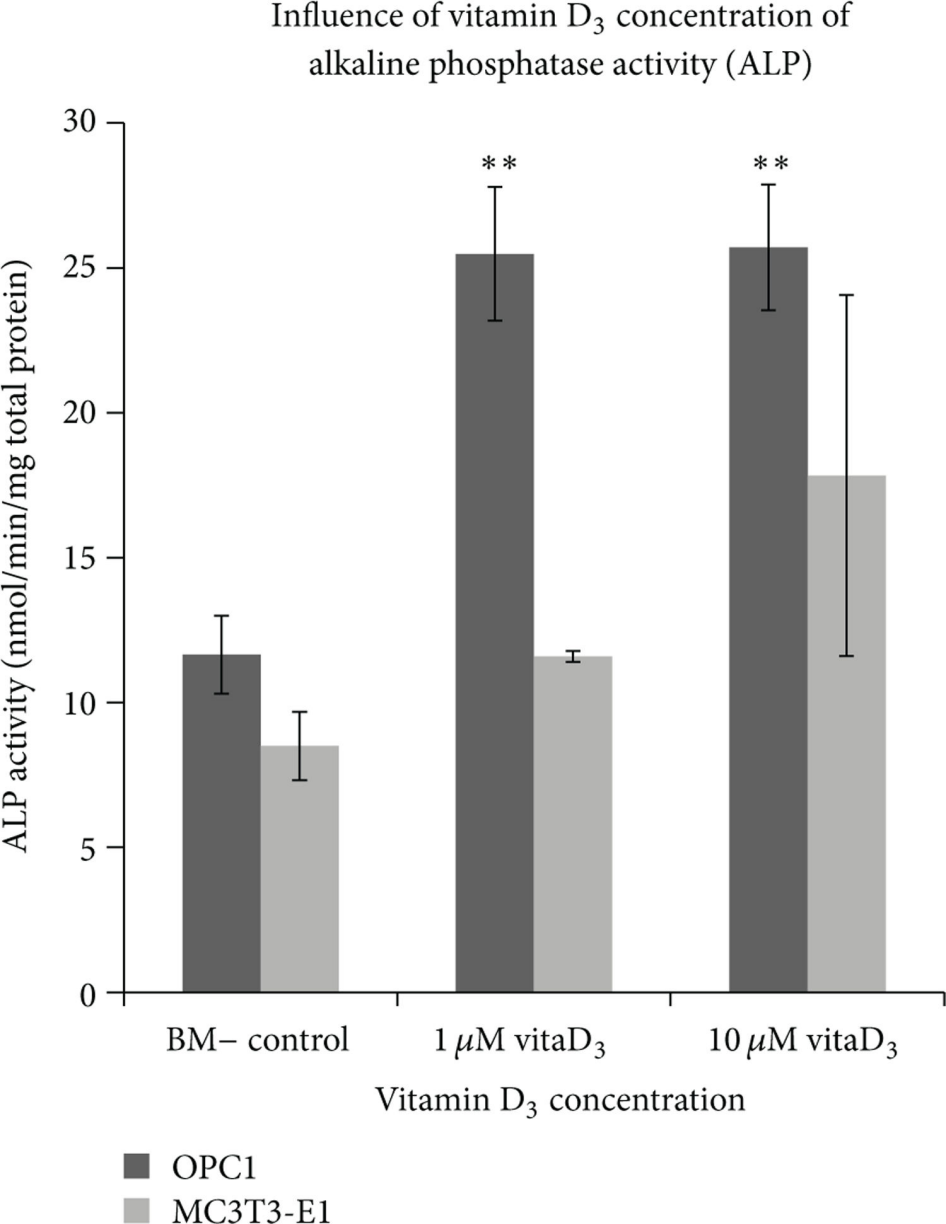
Alkaline phosphatase activity (ALP) of osteoblast precursor OPC1 and MC3T3-E1 after 10 days in culture after being treated with or without vitamin D_3_ (BM− control). After 10 days there was not a significant effect (*P* = 0.2166) on ALP activity between treatment with either 1 *μ*M or 10 *μ*M vitamin D_3_; however, there was an overall statistically significant increase (*P* = 0.0026) in ALP activity between vitamin D_3_ treated cultures and controls.

**Figure 2:**
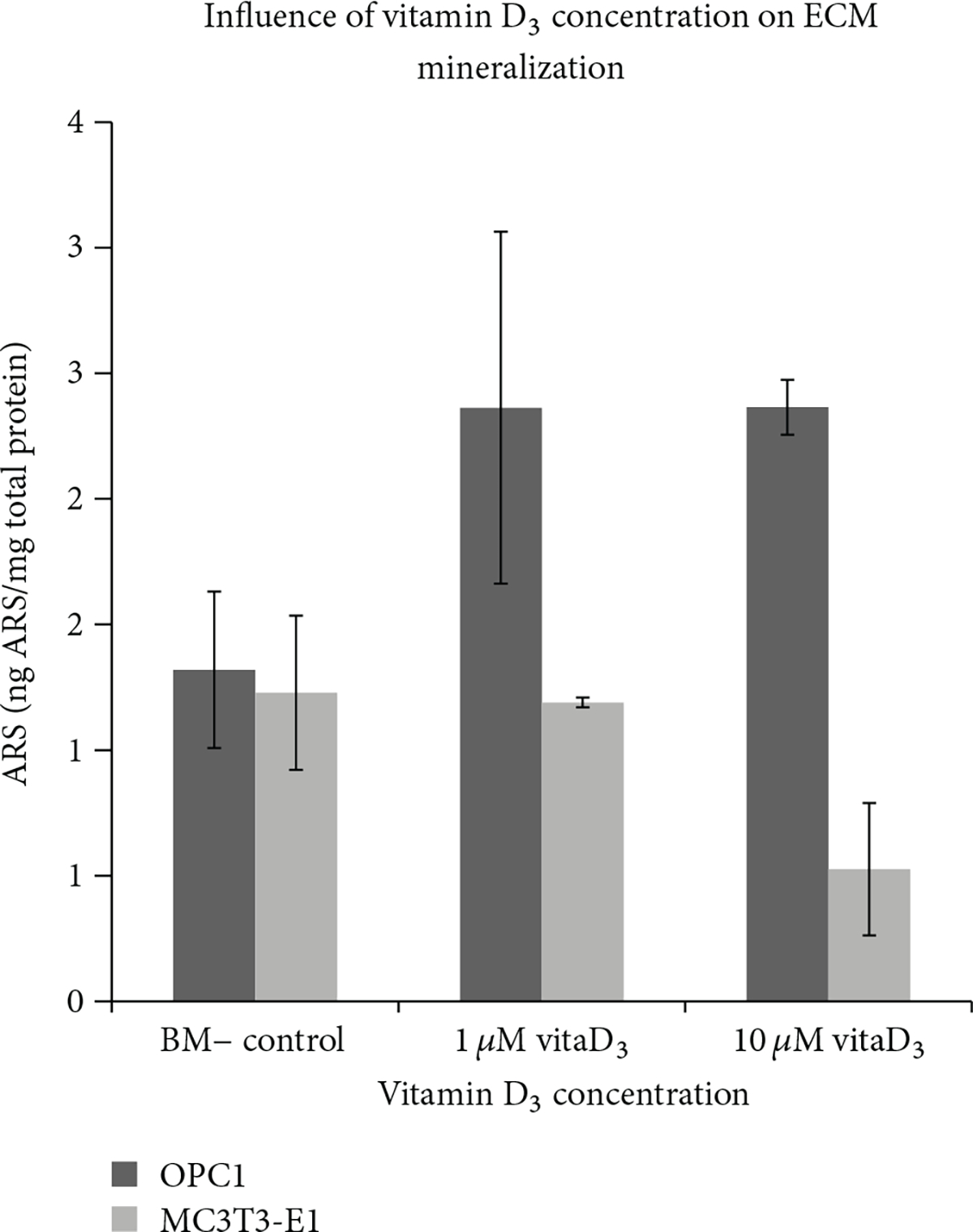
Semiquantification of matrix mineralization was conducted by taking the optical density (resolution = 450 nm) of the ARS extracted from each treatment group. Vitamin D_3_ at both 1 *μ*M and 10 *μ*M dosages did not have a statistically significant effect on the ECM mineralization of OPC1 (*P* =0.2881) or MC3T3-E1 (*p* = 0.1101) after 10 days, compared to the control group.

As described, in order to determine the optimal dose of vitamin D_3_, OPC1 and MC3T3-E1 cells were cultured at a seeding density of 5.0 × 10^4^ in quadruplicates with 1 or 10 *μ*M vitamin D_3_in standard bone medium (BM−)orinaBM− vehicle control containing the equivalent amount of ethanol for 10 days in 12-well plates. After 10 days, two wells from each group were prepared for ALP assay, and two wells were stained with ARS to assess ECM calcium deposition. OPC1 treated with vitamin D_3_ at either concentration had a statistically significant (*P* < 0.001) increase in ALP activity compared to that of the control (Figure 1). There was no statistically significant difference (*P* > 0.05) between experimental groups containing either 1 *μ*M or 10*μ*M vitamin D_3_. There was also no statistically significant difference (*P* = 0.2388)in ALP activity between MC3T3-E1 treated with either 1 *μ*M or 10 *μ*M vitamin D_3_ compared to that of the control. However, in the wells treated with 10 *μ*M vitamin D_3_, we observed a variable effect on ALP activity and an adverse effect on cell viability, which was observed microscopically throughout the experiment (Figure 2). There was not a significant increase in calcium deposition in ECM in either cell lines compared to the ethanol vehicle control (*P* > 0.05 ).

### 3.2. Influence of Vitamin D_3_ and 1,25OH_2_D_3_ on Osteoprecursors

#### 3.2.1. Antiproliferative Effects.

**Figure 3:**
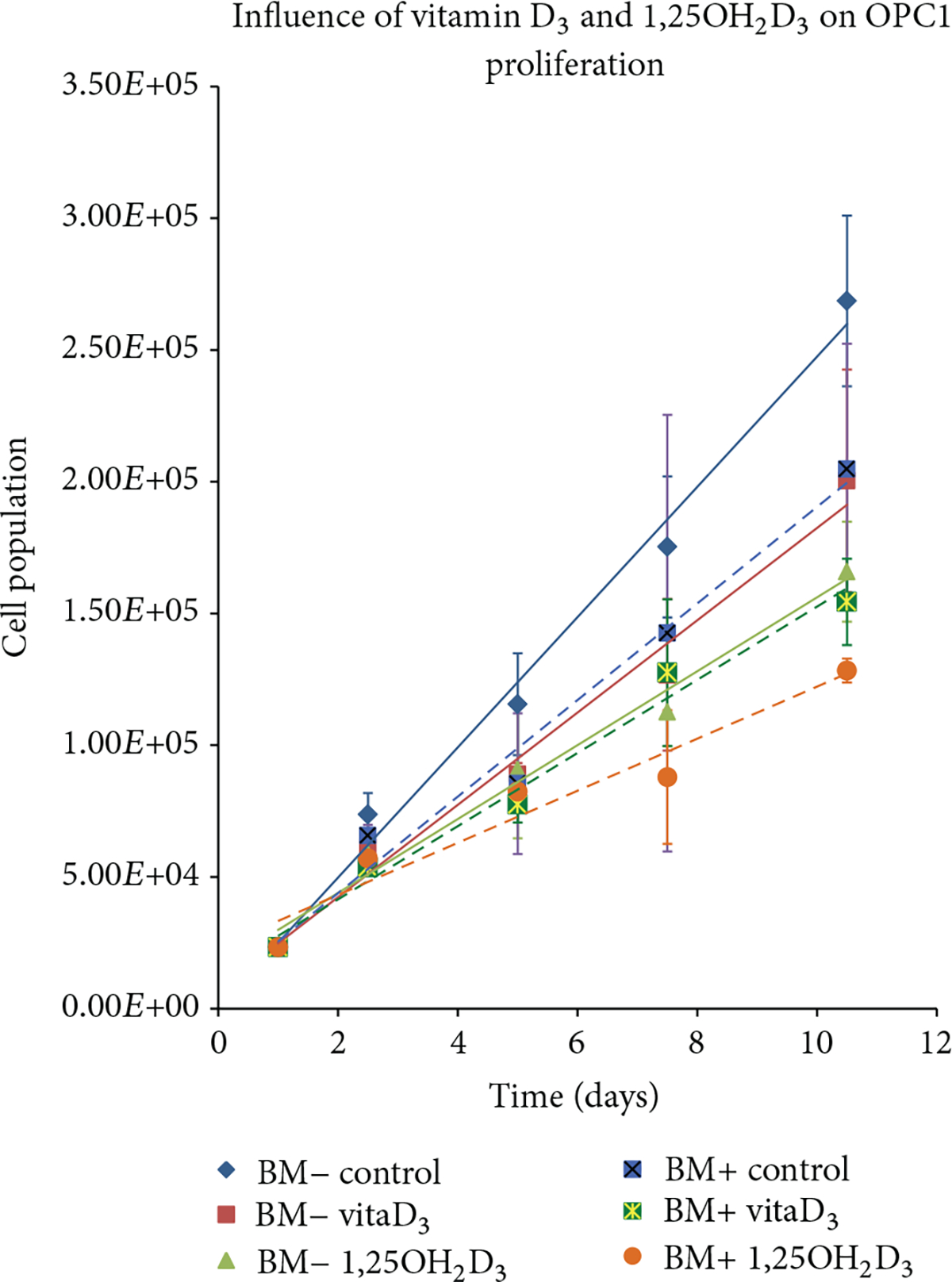
Absolute proliferation of OPC1 cells maintained in six types of tissue culture medium over three two-week periods (*n* = 24). At day 5, the OPC1 cell counts in the wells containing vitamin D metabolites ± osteogenic factors (BM+) were statistically lower than the negative control (BM−). OPC1 treated with BM+ and 1,25OH_2_D_3_ experienced the largest statistically (*P* < 0.001) antiproliferative effect. By day 10, OPC1 treated with BM+ and active 1,25OH_2_D_3_ remained around 50% of the BM− cellular confluency. The effect of vitamin D_3_ was similar to 1,25OH_2_D_3_ in both groups of media. Means ± SEM are shown with linear trendlines in the same color scheme for each experimental group.

**Figure 4:**
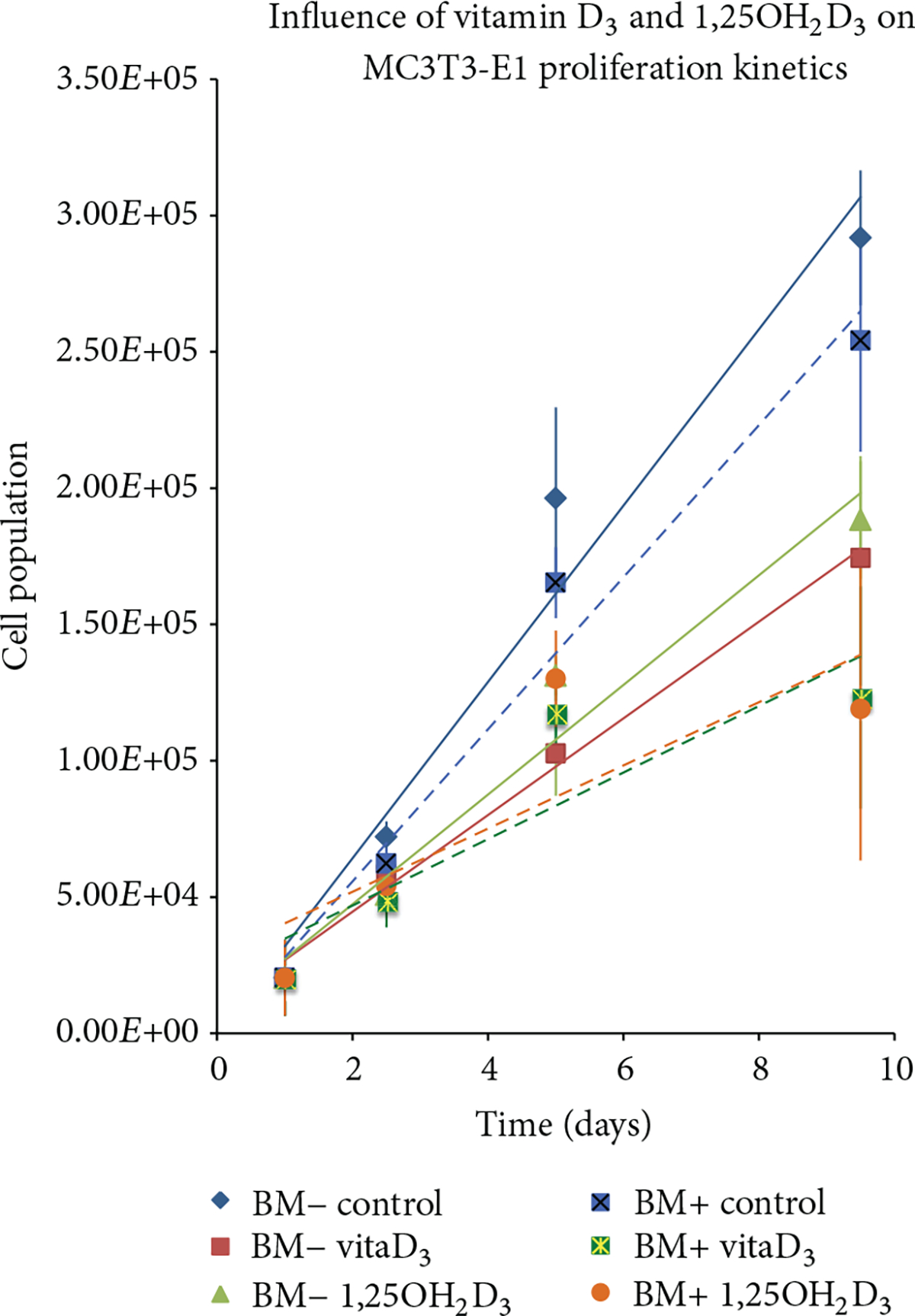
Absolute proliferation of MC3T3-E1 cells maintained in six types of tissue culture medium in duplicates over three two-week periods and assayed in duplicates (*n* = 12 ). Two types of media were used, one with osteogenic factors (BM+) and one without (BM−). At day 5, the MC3T3-E1 cell counts in the wells containing vitamin D metabolites ± osteogenic factor were statistically lower than the negative control (BM−). At day 10, the OPC1 cell counts in the wells containing vitamin D metabolites ± osteogenic factors (BM+) were also statistically lower (*P* < 0.001) then than the negative control (BM−) and remained at around 50% of the BM−cellular confluency. MC3T3-E1 cells treated with BM+ and vitamin D_3_ were indistinguishable from 1,25OH_2_D_3_ in that both experienced the largest statistically (*P* < 0.001 ) antiproliferative effect. Means ± SEM are shown with linear trendlines in the same color scheme for each experimental group.

As described, OPC1 and MC3T3-E1 were cultured for two weeks with either 10 *μ*M vitamin D_3_ or 10 nM 1,25OH_2_D_3_ with (BM+) or without osteogenic factors (BM−) and then compared to BM− and BM+ ethanol vehicle controls. Proliferation kinetics were quantitatively compared after conducting a CVS assay as a function of time. The CVS proliferation assay indicated a statistically significant inhibition of cell proliferation in OPC1 treated with 10 *μ*M vitamin D_3_ in the same manner as cells treated with 10 nM 1,25OH_2_D_3_, compared to that of the BM− and BM+ ethanol controls (shown as absolute cell populations in Figure 3). Groups containing BM+ overall, had lower proliferation rates than with BM− groups; however groups treated with BM+ containing either vitamin D_3_ or 1,25OH_2_D_3_ were significantly lower than the BM− groups. Growth kinetics of MC3T3-E1 cultured with BM+ and 10 *μ*M vitamin D_3_ were indistinguishable from cells cultured in BM+ containing 10 nM 1,25OH_2_D_3_ (shown as absolute cell populations in Figure 4). Results were consistent for both absolute and percentage based cell population analyses.

#### 3.2.2. ECM Maturation.

**Figure 5:**
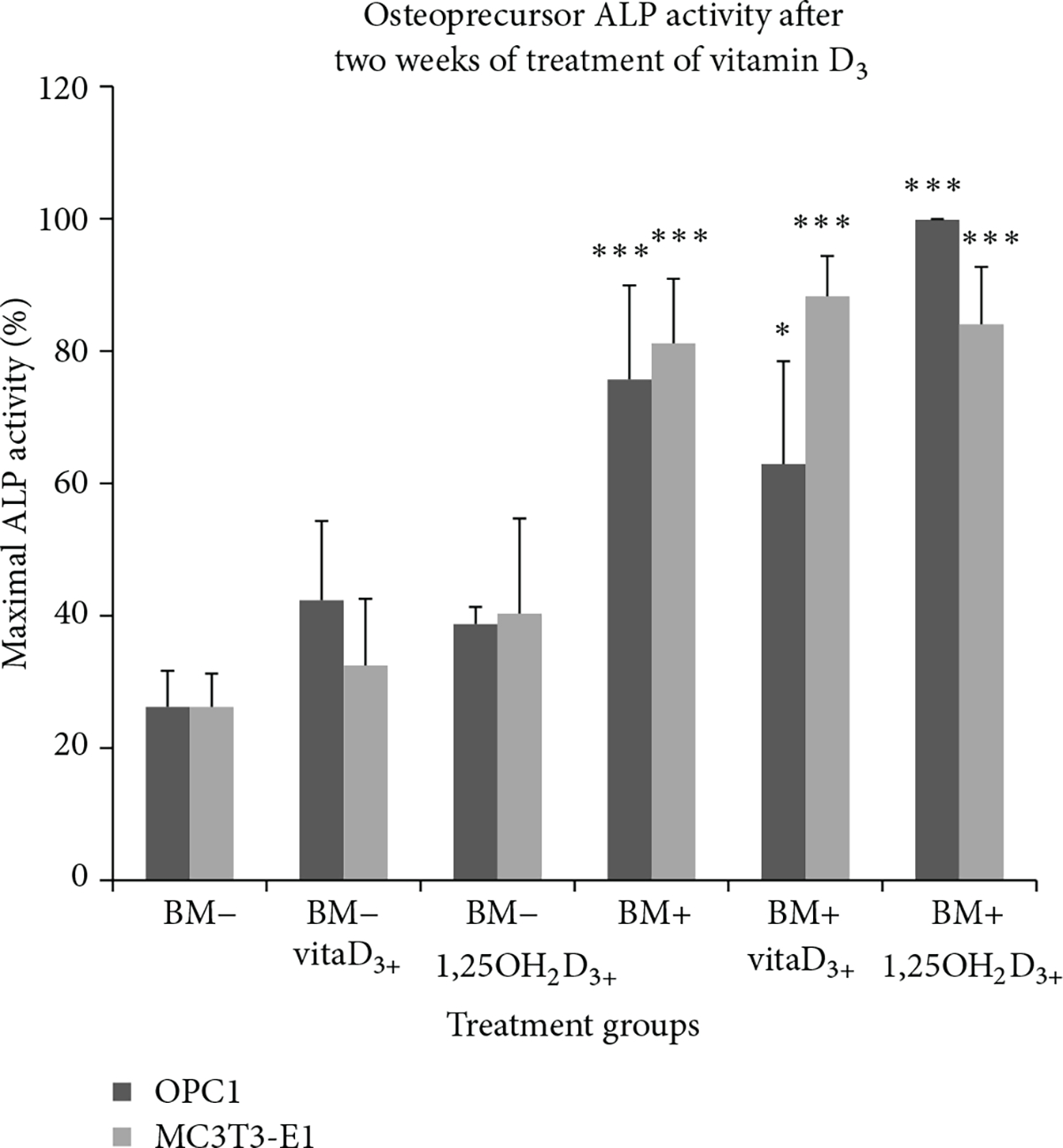
ALP activity of OPC1 and MC3T3-E1 cells maintained in six types of tissue culture medium aggregated over three two-week periods (*n* = 12). After two weeks, the OPC1 ALP activity of 1,25OH_2_D_3_ and osteogenic factors (BM+) treated cells were statistically greater when compared to the negative control (BM−). ALP activity of OPC1 treated with vitamin D_3_ and BM+ was significantly higher than BM− but not higher than the BM+ control containing ethanol vehicle. All cellular groups containing BM+ had a significantly higher ALP activity compared to the control within the MC3T3-E1 cell line. MC3T3-E1 response to vitamin D_3_ was indistinguishable from the response to 1,25OH_2_D_3_.

Increased ALP activity was directly associated with osteoblast function and matrix maturation during the active stages of bone development (Figure 5). Treatment of OPC1 cells with standard bone medium (BM−) and both vitamin D_3_ and 1,25OH_2_D_3_ stimulated a 2- to 3-fold increase in ALP activity after two weeks relative to the parallel control culture, while treatment with osteogenic medium (BM+) containing 1,25OH_2_D_3_ stimulated a 5-fold increase in ALP activity in comparison to BM− and a 20% increase in comparison with the BM+ control (Table 1). However, treatment of MC3T3-E1 cells with BM− containing either vitamin D_3_ or 1,25OH_2_D_3_ showed a slight increase in ALP activity, while BM+ containing either vitamin D metabolite was increased by 20% when compared to the parallel control and a 70 to 80% increase when compared to the BM− control (Table 1).

#### 3.2.3. ECM Mineralization.

**Figure 6:**
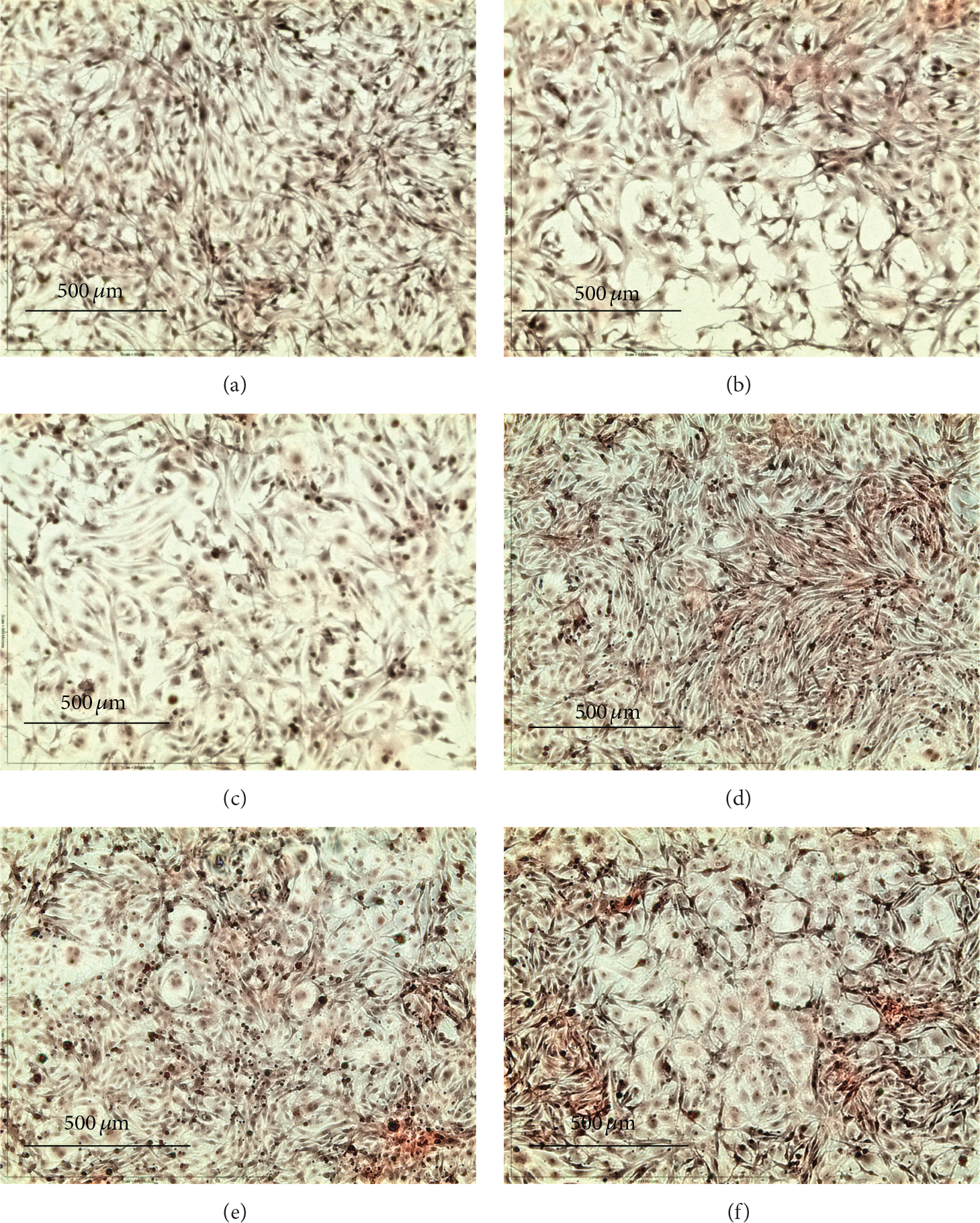
Osteoblast precursor cell lines OPC1 stained with ARS after two weeks in experimental conditions. Images of OPC1 cells without either osteogenic factors (BM−) or vitamin D (ethanol control) (a), BM− with 1 *μ*M vitamin D_3_ (b), and BM− with 10 *μ*M vitamin D_3_ (c) consistently did not absorb a statistically perceptible amount of ARS compared to groups containing osteogenic factors (BM+) (d–f). However, cells cultured with BM+ and1*μ*M vitamin D_3_ (e) and BM+ with 10 *μ*M vitamin D_3_ (f) had a statistically significant (*P* < 0.05 ) amount of calcium associated with ECM mineralization compared to the BM+ control (d). Magnification = ×10, scale = 500 *μ*m.

**Figure 7:**
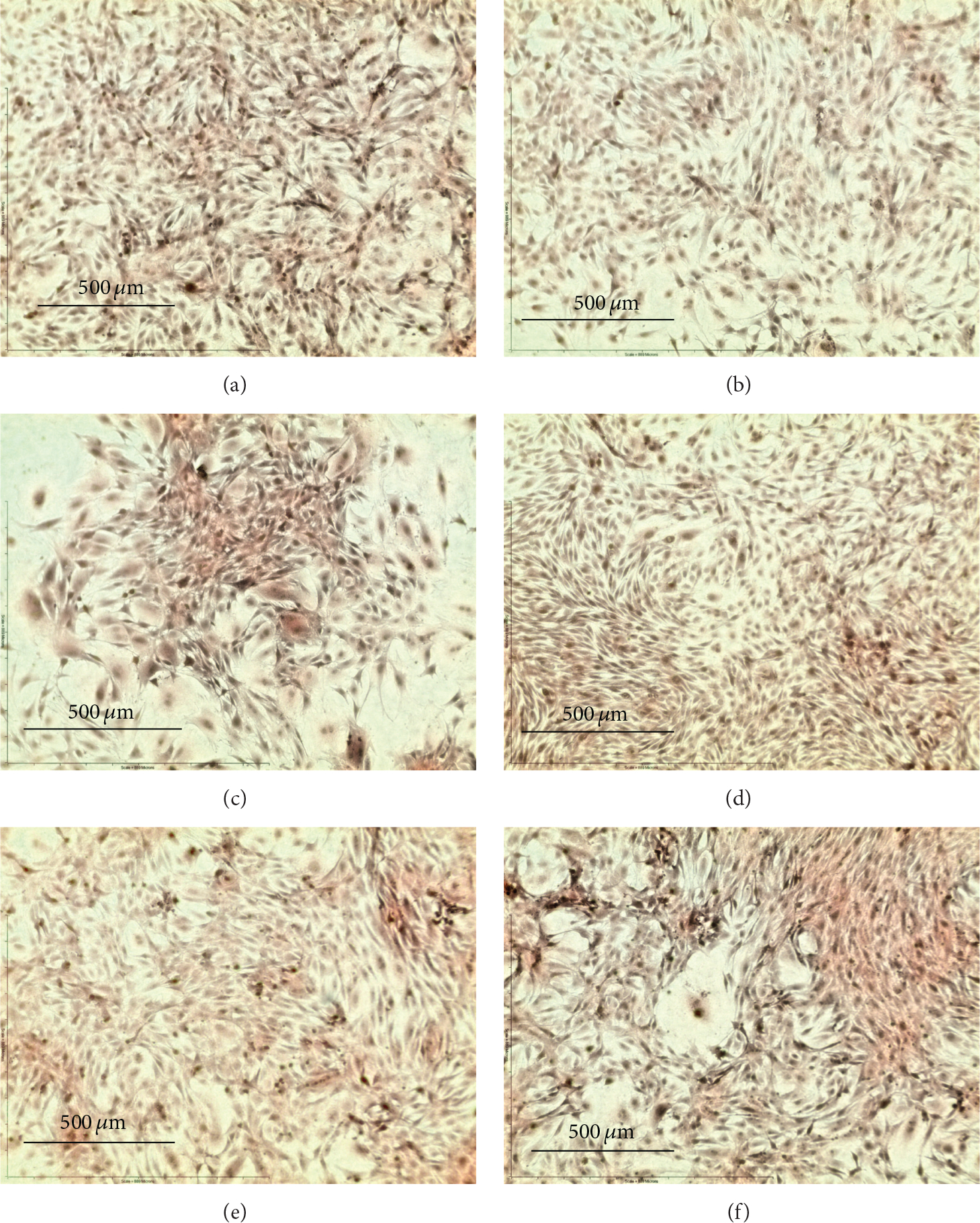
Osteoblast precursor cell line MC3T3-E1 stained with ARS after two weeks within the experimental treatment groups. MC3T3-E1 cells without either osteogenic factors (BM−) or vitamin D (ethanol control) (a), BM− with 1 *μ*M vitamin D_3_ (b), and BM− with 10 nM 1,25OH_2_D_3_ (c) consistently did not absorb a significant amount of ARS compared to groups containing osteogenic factors (BM+) (d–f). Cells cultured with vitamin D_3_ and BM+ (e) were statistically significant compared to the negative control (a) but were not comparable with the parallel control (d). However, cells cultured with BM+ and 10 nM 1,25OH_2_D_3_ (f) had a statistically significant (*P* = 0.0055 )amount of calcium associated with ECM mineralization compared to the BM+ control (d). Magnification = ×10, scale = 500 *μ*m.

**Figure 8:**
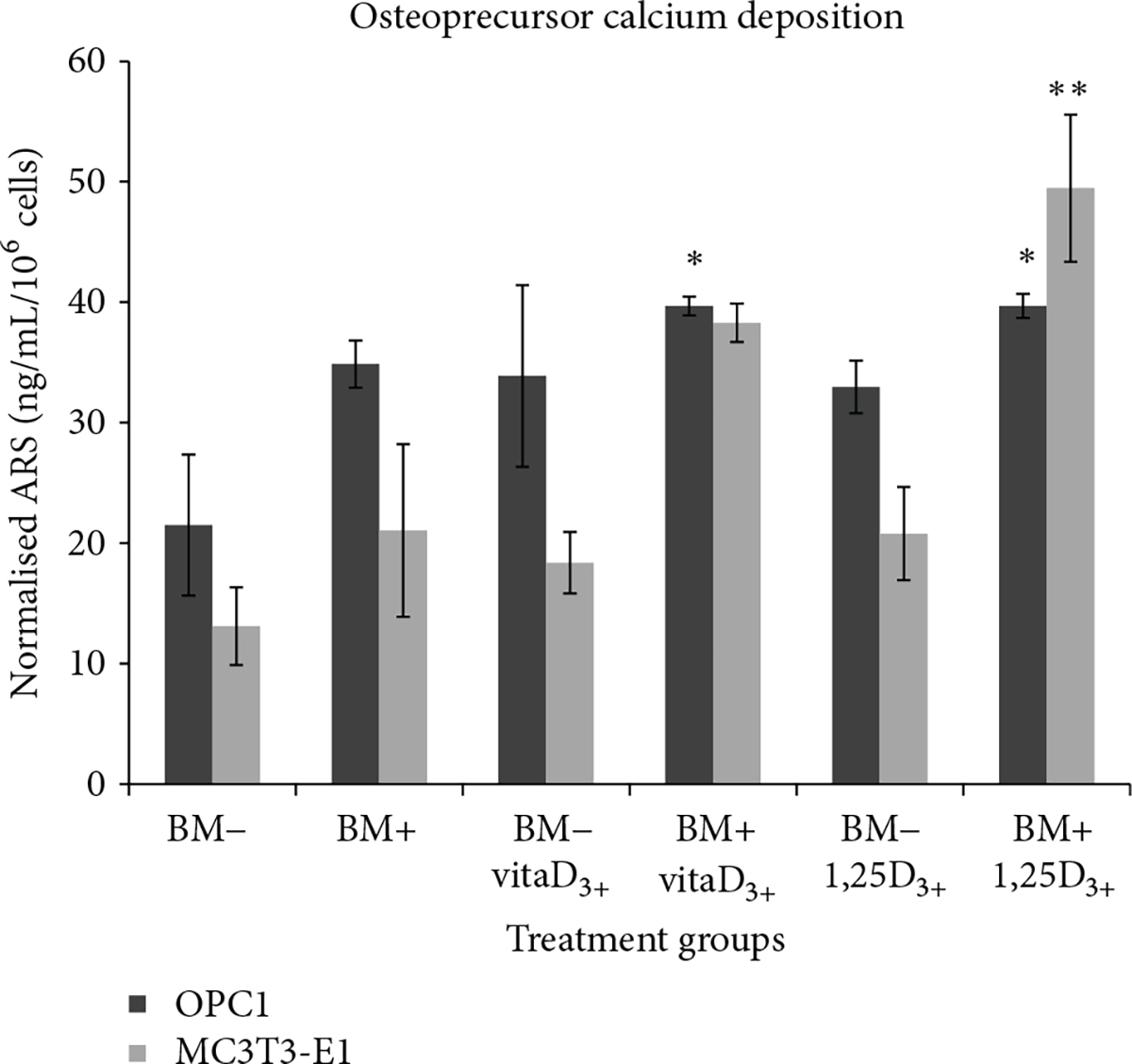
Semi-quantification of matrix mineralization of cultured experimental groups shown in Figures 6 and 7 after two weeks of treatment. OPC1 treated with vitamin D_3_ or 1,25OH_2_D_3_ in BM+ had a statistically significant (*P* < 0.05 ) amount of calcium deposition in comparison to both BM− and BM+ controls. MC3T3-E1 cells cultured with 1,25OH_2_D_3_ in BM+ had a statistically significant (*p* = 0.0055) amount of calcium deposition in comparison with both BM− and BM+ controls.

The ability to mineralize the ECM during bone development provides the functional niche of osteoblasts *in vitro* and *in vivo* [25]. OPC1 groups cultured in BM+ containing vitamin D_3_ (*n* = 8) or 1,25OH_2_D_3_ (*n* = 8) had a statistically larger amount of ECM calcium compared to the BM− (*P* < 0.001) and BM+ (*P* = 0.027) ethanol vehicle controls based on ARS measurements (Figure 6). The optical density of the OPC1 cell culture (measured in normalized ARS) increased by 100% relative to the negative control (BM−)and by about 10% compared to the BM+ parallel control (Figure 8). OPC1 cells treated with BM+ and vitamin D_3_ (39.68 ng/mg/10^6^ cells ± 0.75, *n* = 8) were indistinguishable from cells cultured with BM+ and 1,25OH_2_D_3_ (39.69 mean ± 1.00, *n* = 8 )(*P* < 0.05 ). MC3T3-E1 groups cultured in BM+ containing vitamin D_3_ (*n* = 8 ) or 1,25OH_2_D_3_ (*n* = 8 ) had a statistical greater amount of ECM calcium compared with treatment groups with only BM− (*P* < 0.001). Meanwhile, cultures with BM+ and 1,25OH_2_D_3_ had a statistically greater amount of ECM calcium compared with the BM+ parallel control (*P* = 0.0055) (Figures 7 and 8).

### 3.3. Endogenous 1,25OH_2_D_3_ Synthesis by OPC1.

**Figure 9:**
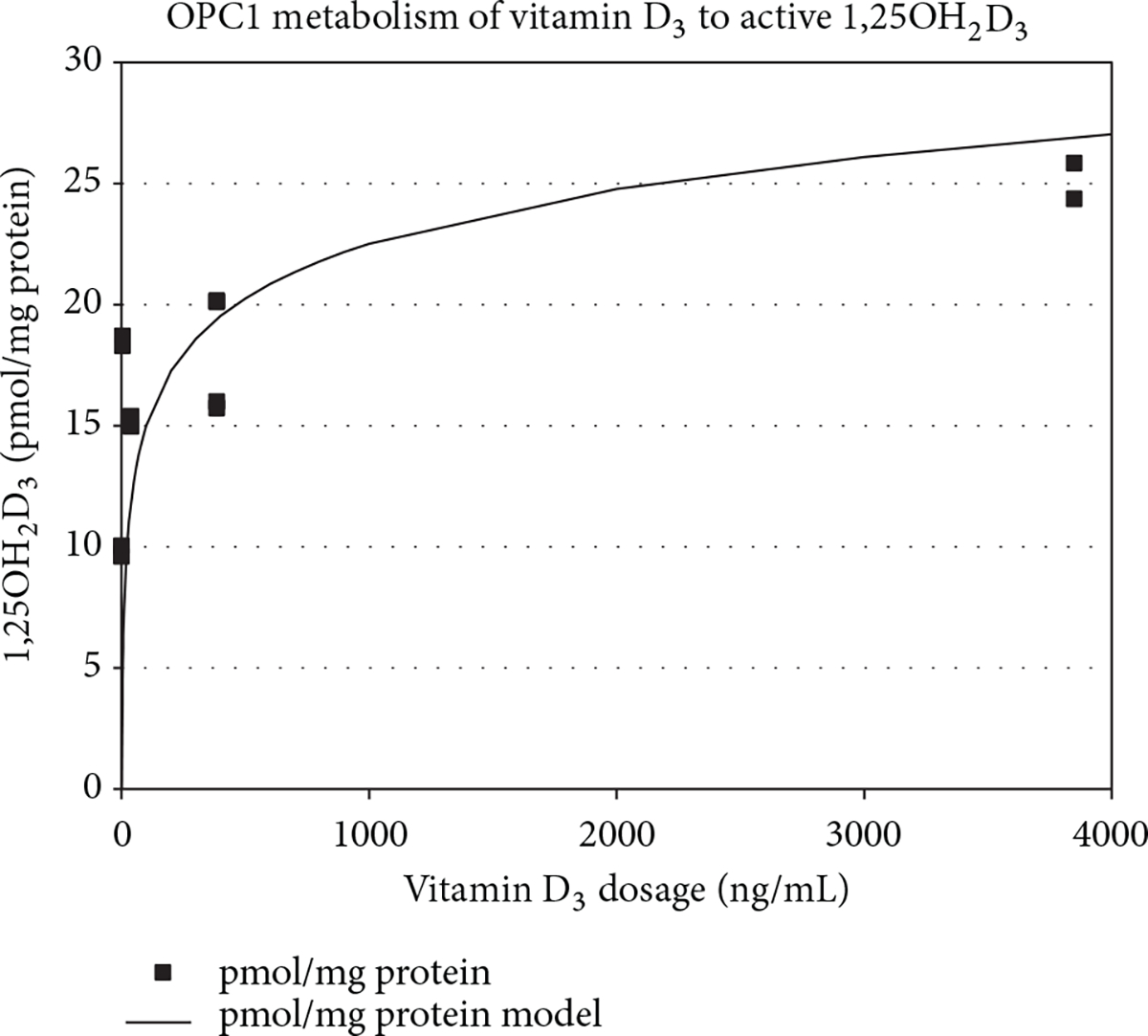
Endogenous production of 1,25OH_2_D_3_ by OPC1 in response to incubation with vitamin D_3_. A 1,25OH_2_D_3_/VDR ELISA was performed to analyze the metabolism of precursor vitamin D_3_ to the hormonally active form. The dose of vitamin D_3_ was controlled and administered at logarithmic concentration levels. A log-base-10 model was applied to the resulting response data.

OPC1 cultures were tested for their ability to synthesize 1,25OH_2_D_3_ from the parental precursor vitamin D_3_. For this dose-response assay, cells were cultured at high density in order to maximize the concentration of secreted 1,25OH_2_D_3_. Low serum conditions were also used and compared with a serum control to account for any potential influence of vitamin D metabolites present in the serum. OPC1 cultured in the presence of vitamin D_3_ produced a detectable level of 1,25OH_2_D_3_ that elevated with increasing concentration. The synthesis of 1,25OH_2_D_3_ in OPC1 correlated logarithmically to the dosage of vitamin D_3_ when applying the log model of (1) (*R*^2^ = 0.9898, *P* = 0.0051, and *m* = 7.23 ng/mL) (Figure 9).

## 4. Discussion

We investigated whether osteoblast precursors have the capacity to convert vitamin D_3_ to 1,25OH_2_D_3_ and examined the potential of vitamin D_3_ to induce 1,25OH_2_D_3_ associated biological activities in osteoblast precursors. The active form of vitamin D, 1,25OH_2_D_3_, is generated from the parental precursor of vitamin D_3_ which requires two sequential hydroxylation steps catalyzed by mitochondrial cytochrome P450 enzymes, CYP27A1, and CYP27B1, respectively [26]. We found that OPC1 is capable of synthesizing 1,25OH_2_D_3_ by administering vitamin D_3_ in a dose-dependent manner and that the influences of vitamin D_3_ on antiproliferation and osteoblast differentiation were very similar to 1,25OH_2_D_3_. It was also found that vitamin D_3_ induced ALP activity and calcium deposition associated with ECM maturation and mineralization during early bone development in a manner that was indistinguishable from groups treated with 1,25OH_2_D_3_. These findings have important implications for bone and vitamin D research because they provide further evidence that local production of active 1,25OH_2_D_3_ can occur in osteoprecursors and that local metabolism of vitamin D_3_ can influence bone development.

Several osteoblast culture systems have demonstrated that cells undergo a temporal developmental sequence that starts with a proliferation phase and results in fully mature osteocyte-like cells embedded in mineralized ECM [5, 27]. There are three stages of *in vitro* bone development that have been characterized by morphology and gene expression. These are (1) a proliferation and ECM biosynthesis stage, (2) an ECM development and maturation stage noted by an increase in ALP activity, and (3) a final stage of ECM mineralization in which hydroxyapatite is organized and deposited [5, 6, 27]. In the first stage, following initial cell seeding, the cells actively proliferate for the first 10 to 12 days of culture, and several genes associated with ECM formation, including type I collagen and osteopontin, are actively expressed and then gradually decline as proliferation rate decreases and ECM maturation begins. We report that both vitamin D_3_ and 1,25OH_2_D_3_ had similar effects on two osteoprecursor cell lines, which influenced the proliferation stage of bone development by significantly hampering the growth rate.

Immediately following the decrease in proliferation, ALP activity is greatly increased. This postproliferative period occurs between days 12 and 18, and it is during this time that the ECM matures and becomes competent for mineralization [5, 6]. Here, ALP activity of untreated osteoprecursors steadily increased, while the groups containing osteogenic factors (BM+) and either vitamin D_3_ or 1,25OH_2_D_3_ increased more than 10-fold over a two-week period of time. While the measurement of increased ALP expression is used as an osteogenic marker as an indication of osteoblast phenotype, its function still remains unclear. It has been postulated to increase concentration of inorganic phosphate, a concept known as the “booster hypothesis” [28]. During the third stage of bone development, ALP expression decreases, and other genes associated with bone mineralization are expressed at maximal level [6, 27, 28]. These bone cell synthesized proteins include osteopontin (OP) and osteocalcin (OC) and are known to interact with the mineralized ECM *in vivo* [6]. OP is expressed in both the proliferation and mineralization periods and downregulated postproliferatively during matrix maturation; however, during proliferation, OP expression reaches only about 25% of its maximal level which peaks between days 16 and 20 during mineralization. In contrast, the calcium binding protein OC, which binds to hydroxyapetite, is expressed only postproliferatively with the onset of nodule formation and is expressed maximally with ECM mineralization *in vivo* and *in vitro* [6]. We saw minimal calcium mineralization after two weeks in untreated cell cultures as well as calcium mineralization of the ECM in cultures that were treated with osteogenic factors (BM+) and vitamin D_3_ or 1,25OH_2_D_3_. However, further studies need to be carried out for a longer duration to see maximal levels of ECM mineralization that are associated with bone development.

The osteoprecursor cultures observed in the present work were nonconfluent cultures with actively dividing cells. Because vitamin D has an antiproliferative effect, it is important to look at its influence on osteoblast precursors after proliferation begins to decrease in order to get a better understanding of how vitamin D affects a confluent cell population. Both OPC1 and MC3T3-E1 cultures treated with vitamin D_3_ or 1,25OH_2_D_3_ did not reach confluency during the 15 day culture period, while the control groups reached confluency at around 10 to 12 days after an initial seeding density of about 6,500 cells/cm^2^. Confluent osteogenic cultures, including MC3T3-E1 cells and human bone marrow derived stromal cells (BMSCs), follow a two-stage developmental process including a 1- to 2-week initiation phase during which cells slowly proliferate, express ALP activity and other bone specific genes, and produce and assemble a collagen matrix and a second maturation phase occurring in weeks 2 to 3 in which ECM mineralization is observed [24]. Mineralization has been demonstrated in BMSC cultures that achieve a minimal ALP activity (~0.25 nmol/min/*μ*g protein or 1.2 nmol/min/10,000 cells) at some point during the 2 to 3 week culture period, albeit some BMSC cultures can produce high levels of ALP *in vitro* without ever mineralizing [24]. Treatments applied here tended to speed up maturation at the expense of proliferation; hence, confluency would be achieved more slowly (or not at all, in this case) relative to control cultures. The effects of vitamin D on ALP activity and mineralization of ECM on confluent OPC1 cultures are currently under way.

One limitation here is that because supraphysiological doses (≥10^-7^ M) of vitamin D_3_ (10 *μ*M) and 1,25OH_2_D_3_ (10 nM) were used to achieve a maximal response, it is unclear how relevant our findings are to the action of lower vitamin D_3_ levels within the physiologic range. *In vivo*, doses of vitamin D_3_ as low as 10 *μ*g (400IU) have been shown to stimulate bone mineral augmentation in adolescents [29], and the administration of physiological doses of 1,25OH_2_D_3_ has been shown to cure vitamin-D-dependency rickets I [30]. Our findings indicated that, for an *in vitro* system, supraphysiological doses had a positive effect on bone maturation and mineralization and a negative response in cellular proliferation.

Another limitation is that an insufficient amount of RNA was obtained from the groups containing vitamin D_3_ and 1,25OH_2_D_3_ to permit simultaneous analysis by qRT-PCR of multiple primers. This was due to the fact that both vitamin D_3_ and 1,25OH_2_D_3_ inhibited proliferation, which lead to a much smaller population size in comparison to the controls.

Advancements in the design of biomedical engineering technologies, especially those supporting regenerative medicine through cell and tissue engineering, include the use of precursor cell lines which can consistently be manipulated to develop neotissue. The OPC1 cell line investigated here maintained consistent proliferation, ECM synthesis, and boney maturation, albeit during an abbreviated two-dimensional study. Future investigations will recreate the culture scenarios in a three-dimensional, cell-biomaterial construct with concomitant mechanical stimulation through the application of a novel bioreactor [31]. This approach will establish a surrogate living tissue model for pharmacokinetic assessment of the dose-response relationship building off of the foundation described here.

## Figures and Tables

**Table 1:** ALP activity of OPC1 and of MC3T3-E1 cells maintained in six types of tissue culture medium experimental groups over three two-week periods (*n* = 12). After two weeks, both the OPC1 and MC3T3-E1 ALP activity values of 1,25OH_2_D_3_ and osteogenic factors (BM+) treated cells were statistically greater than the negative control (BM−). ALP activity of OPC1 treated with vitamin D_3_ and BM+ was significantly higher than BM− but not higher than the BM+ control containing ethanol vehicle. All groups containing BM+ had a statistically greater ALP activity compared to the control in the MC3T3-E1 cell line. Also, the MC3T3-E1 response to vitamin D_3_ treatment was indistinguishable from the response to 1,25OH_2_D_3_ treatment.

Maximum ALP activity (mean % [SEM])					
Cell type	Experimental group	Culture time (days)			
		2	5	9/10	14/15

OPC1	BM− control	6.95 [4.29]	12.49 [2.53]	15.73 [4.64]	21.39 [6.16]
	BM− vitaD_3_	7.59 [4.60]	21.27 [8.72]	22.45 [6.60]	34.87 [11.29]
	BM− 1,25OH_2_D_3_	6.15 [2.48]	11.21 [0.42]	15.63 [2.48]	31.90 [7.05]
	BM+ control	8.99 [4.87]	19.20 [5.92]	40.23 [4.71]	69.11 [12.02]
	BM+ vitaD_3_	9.63 [5.42]	11.11 [2.86]	21.48 [10.74]	62.90 [15.56]
	BM+ 1,25OH_2_D_3_	5.07 [1.04]	18.77 [7.70]	54.10 [22.78]	99.81 [0.14]

MC3T3-E1	BM− control	1.41 [0.79]	4.44 [1.37]	5.31 [1.39]	26.21 [5.04]
	BM− vitaD_3_	2.72 [0.53]	7.83 [3.13]	13.46 [3.81]	32.44 [10.10]
	BM− 1,25OH_2_D_3_	2.82 [1.50]	9.18 [3.80]	13.79 [6.00]	40.29 [14.42]
	BM+ control	4.13 [2.33]	11.94 [7.78]	22.67 [6.74]	81.17 [9.74]
	BM+ vitaD_3_	2.25 [0.90]	15.76 [8.26]	24.95 [7.42]	88.24 [6.12]
	BM+ 1,25OH_2_D_3_	2.55 [1.37]	13.19 [5.03]	42.76 [13.35]	84.03 [8.71]
